# A preliminary study on changes in heat shock protein 70 levels induced by *Fusarium* mycotoxins in rats: in vivo study

**DOI:** 10.1007/s12550-021-00425-z

**Published:** 2021-03-04

**Authors:** Dániel J. Kócsó, Omeralfaroug Ali, Melinda Kovács, Miklós Mézes, Krisztián Balogh, Mariam L. Kachlek, Brigitta Bóta, Yarsmin Y. Zeebone, András Szabó

**Affiliations:** 1 Institute of Physiology and Nutrition, Department of Physiology and Animal Health, Hungarian University of Agriculture and Life Sciences, MTA-KE-SZIE Mycotoxins in the Food Chain Research Group, Kaposvár Campus, Kaposvár, Hungary; 2 Institute of Physiology and Nutrition, Department of Physiology and Animal Health, Hungarian University of Agriculture and Life Sciences, Kaposvár Campus, Kaposvár, Hungary; 3 Institute of Physiology and Nutrition, Department of Feed Safety, Hungarian University of Agriculture and Life Sciences, Gödöllő Campus, Gödöllő, Hungary

**Keywords:** Rat, Fumonisin B_1_, Deoxynivalenol, Zearalenone, Heat shock protein, Multitoxic effect

## Abstract

The heat shock protein (Hsp70) level was assessed after 14 days of oral gavage-exposure to fumonisin B_1_ (FB_1_: 150 µg/animal/day), deoxynivalenol (DON: 30 µg/animal/day) and zearalenone (ZEN: 150 µg/animal/day), alone or in combinations (in additive manner: FD = FB_1_ + DON, FZ = FB_1_ + ZEN, DZ = DON + ZEN and FDZ = FB_1_ + DON + ZEN) in the liver, kidneys and lung of 24 adult male Wistar rats (*n* = 3/group). The liver was the most responsive tissue, as compared with kidney and lung. Except of DZ-treatment, mycotoxins elevated the Hsp70 levels in livers. The highest Hsp70-levels (≈ twofold) were in the DON, FD, FZ and FDZ treatments (additive effects). In the kidney, alterations (↑ ≈ twofold) were detected in ZEN, FD, FZ and DZ treatments. The least responsive organ was the lung (↑ only in FDZ, antagonistic effect). DON and ZEA exposures have altered the reduced glutathione concentration (↓) and glutathione peroxidase activity (↓) in the blood serum. The serum malondialdehyde level increased only after exposure to FD (synergistic effect), as compared with the DZ group (antagonistic effect). When the blood clinical chemistry was assessed, significant alterations were in alanine aminotransferase (80% increase in FDZ, antagonistic effect) and total protein (↓ ZEN). Results varied according to the organ, toxin type and interactions. Furthermore, oxidative stress was not the only key player behind the Hsp70 increase, in which another mechanism is suggested.

## Introduction

*Fusarium* mycotoxins, namely deoxynivalenol (DON), fumonisins (FBs) and zearalenone (ZEN), are frequently occurring in animal feed (Gruber-Dorninger et al. 2019). The effects of mycotoxins are exerted through different intracellular signal transduction mechanisms. DON has various cytotoxic mechanisms documented, including the bond formation with the 60S subunit of the eukaryote ribosome, inhibition of the peptidyl transferase activity, activation of different cellular kinases (e.g. c-Jun N-terminal kinases, JNK) and induction of the transcription factor nuclear factor-kappa B (NFƙB) expression (Pestka 2007). FB_1_ blocks the enzyme sphinganine N-acetyltransferase activity, and consequently, disrupts the sphingolipids metabolism (Norred et al. 1996) and affects cell membrane lipids (Szabó et al. 2019). ZEN has an oestrogen analogue structural; therefore, ZEN expresses a high affinity to cytosolic 17β-oestradiol receptors (ERα and ERβ), exerting both agonistic and antagonistic effects at these receptors (Zinedine et al. 2007).

*Fusarium* mycotoxins vary in disruption level on the cellular redox balance system; ZEN > FB_1_ > DON (El Golli-Bennour and Bacha 2011). The redox sensors, namely the heat shock proteins (Hsps), have remarkable cytoprotective mechanisms; they act as molecular chaperones (Ikwegbue et al. 2018). The 70-kDa heat shock factors (Hsp70s) production relies on the bond formation with the trimerization domain of type 1-heat shock transcription factor (HSF-1) (Fehrenbach and Northo 2001). In response to the different lethal stimuli, Hsp70 can increase the cellular survival chance by aiding the protein assembling and folding through mitigating the mitochondrial damage, nuclear fragmentation and stress-induced caspase-cascade (Jee 2016; Mosser et al. 1997). The base of this anti-apoptotic effect is the so-called Hsp70-mediated modulation of the proteasome.

Up to date, only a few in vitro and in vivo studies investigated the two mycotoxins interaction on rodents (Ren et al. 2016; Liang et al. 2015; Sun et al. 2015; Szabó-Fodor et al. 2015; Kouadio et al. 2013; Tajima et al. 2002; Groten et al. 1998; Pestka et al. 1987; Forsell et al. 1986). Scares studies are available on the ternary mycotoxin interactions in rodents (Szabó-Fodor et al. 2018, 2015; Szabó et al. 2018; Tajima et al. 2002) but none investigated the Hsps. Our study is a part of the larger in vivo study published by Szabó et al. (2018) and aimed to investigate the individual and combined mycotoxin (FB_1_, DON and ZEN) effects, with a particular interest to the intracellular defence mechanisms, namely Hsp70 and redox system.

## Materials and methods

### Animals, feeding and experimental protocol

Adult, male Wistar Crl:WI BR rats (8 weeks of age) were enrolled in the study and were kept in metabolic cages (Tecniplast, Castronno, Italy) individually. The animals (*n* = 33/group, 8 groups, total *n* = 324) were fed Ssniff R/M-Z + H feed (Ssniff GmbH, Soest, Germany; 19% crude protein, 3.5% crude fat, 3.6% crude fibre, 16.4 MJ/kg gross energy). The rats were kept in a 12-h light and 12-h dark daily rhythm, at 20 °C in a rodent room. The relative air humidity was 50%. Feed was offered ad libitum, and feed intake was measured daily.

Mycotoxin treatment was set as follows for individual toxins: control (C): toxin free, FB_1_ (F): 5 mg/kg diet (150 µg/animal/day), DON (D): 1 mg/kg diet (30 µg/animal/day) and ZEN (Z): 0.5 mg/kg diet (15 µg/animal/day). The binary (FD, FZ, DZ) and ternary (FDZ) mixtures of the toxins contained the same mycotoxin concentrations in an additive manner. The mycotoxins were purchased from Sigma-Aldrich (Schnelldorf, Germany), and stock solutions were prepared with double-distilled water.

The mycotoxin concentration of the standard solutions was controlled daily with LC-MS (Shimadzu 2020 (Shimadzu, Kyoto, Japan) instrument equipped with a Phenomenex (Phenomenex, Torrance, USA) Kinetex 2.6 µm C18 100 Å 150 × 3 mm column). Internal standards were used in all instances, as purchased from Romer Labs (U-[^13^C34]-Fumonisin B_1_(cat. no.: 10002806), U-[^13^C15]-deoxynivalenol (cat. no.: 10000332) and U-[^13^C18]-zearalenone (cat. no.: 10002816). The standard solutions were used if the recovery was between 100 ± 0.1% (since this was only a “quality control,” data are not shown of this measurement).

The solutions contained the daily toxin dose in a volume of exactly 1 ml, and this solution was administered as a single gavage dose orally. For the control animals, 1 ml of double-distilled water was dosed. After 14 days of exposure, the animals were sacrificed by cervical dislocation and immediately dissected. Liver, kidney, lung and blood (plasma) samples were collected and stored frozen (− 80 °C and − 20 °C, respectively) until analysis.

The Food Chain Safety and Animal Health Directorate of the Somogy County Agricultural Office authorised the experimental protocol, under the allowance number SOI/31/1679-11/2014.

### Western blot analysis

Liver, kidney and lung tissue samples (*n* = 33/group) were homogenised in 500 µl lysis buffer (1% NP40, 1% Na-deoxycholate, 0.1% SDS, 15 mmol/L NaCl, 10 mmol/L phosphate buffer, 2 mmol/L EDTA, 2 mg/ml aprotonin, 0.5 mg/ml leupeptin, 2 mmol/L Na-vanadate, 20 mmol/L NaF, 0.5 mmol/L DTT, 1 mmol/L PMSF) for 3 min. Subsequently, the cell lysate was centrifuged (17,000 g, 30 min, 4 °C) and the pellet was collected. Total protein concentration of the samples was determined with the BCA™ Protein Assay KIT (Thermo-Fisher, Budapest, Hungary), in which 35 µg protein/sample quantities were loaded onto 10% SDS-polyacrylamide gels (30% Acrylamide/Bis-acrylamide, 1.5 mol/L Tris (pH 8.8), 100 g/L SDS, 100 g/L APS, TEMED), which were then transferred to nitrocellulose (0.45 µm) membrane. The membranes were washed with TBS-T (1 × TBS pH 7.6, 0.1% Tween 20) for 3 × 5 min, then blocked in 1 × PBS (PBS, 10 × phosphate-buffered saline) containing 5% non-fat, dried milk powder, 1% BSA and 0.1% Tween-20. Subsequently, the membranes were incubated with the primary Anti-Hsp70 antibodies (1:1000; Merck-Sigma Cat. No. SAB4200714) at 4 °C for ~ 12 h. As internal control, Anti-β-actin antibodies (1:10,000; Sigma, Budapest, Hungary) were used. After another 3 × 5-min washing with TBS-T (pH 7.5), horseradish peroxidase- (HRP-) conjugated secondary antibodies were used in 1:10,000 dilution (Biomarker, Budapest, Hungary) to quantify the binding of the primary antibodies. After a repeated 3 × 5-min TBS-T washing, the light emission of the blotted proteins was ensured with a WesternBright Enhanced Chemiluminescent HRP substrate detection system (Biomedica, Budapest, Hungary) and detected either on CL-XPosure clear-blue X-ray films or by the use of a FluorChem Q Imaging system imaging program (ProteinSimple, Santa Clara, CA, USA). Densitometric analysis of the thus obtained chemiluminescent signals was performed using the ImageJ (Rasband 1997) software. The data were presented by pixel density in arbitrary units ± SEM (*P* < 0.05).

### Serum clinical chemistry

The concentrations of plasma total protein, albumin, and urea, as well as the activities of aspartate aminotransferase (AST), alanine aminotransferase (ALT), were determined in a veterinary laboratory (Vet-med Laboratory Budapest, Hungary), using a Roche Hitachi 912 Chemistry Analyzer (Hitachi, Tokyo, Japan) with commercial diagnostic kits (Diagnosticum Ltd., Budapest, Hungary).

### Lipid peroxidation and antioxidant system

For the determination of lipid peroxidation, blood plasma samples were stored at − 80 °C until analysed. Lipid peroxidation was determined by the quantification of malondialdehyde (MDA) levels by the 2-thiobarbituric acid method (Placer et al. 1966) in the blood plasma and in 1:9 homogenates of tissue samples in physiological saline. Among the components of the antioxidant system, the concentration of reduced glutathione (GSH) was measured in the blood plasma and in the 10,000 g supernatant fraction of tissue homogenates by the method of Sedlak and Lindsay (1968) and the activity of glutathione peroxidase (GPx) according to Lawrence and Burk (1978). GSH concentration and GPx activity were calculated to protein content which was determined by the biuret method in the blood plasma (Weichselbaum 1948) and with Folin phenol reagent in tissue homogenates (Lowry et al. 1951).

### Statistical analysis

For the measured parameters (Hsp70 expression, antioxidant parameters, clinical chemical parameters and oxidative capacity), analysis of variance (ANOVA) was carried out by using IBM Statistics software version 20 (2009). To identify the differences between single treatments, the post hoc Tukey’s multiple comparison test was used. The test was considered significant when the probability was lower than 0.05 (*P* < 0.05). Furthermore, according to the *P*-value, the significance level between intergroup was classified into significant and highly significant: 0.05 and 0.01, respectively.

If a significant difference was detected between two or multiple groups, the Bliss independence method was applied to ascertain possible, mycotoxin-treatment associated interactions (Bliss 1939). Results are shown in a textual form, merely for the cases where ANOVA also provided significant inter-group differences.

## Results

### Liver Hsp70 levels

**Fig. 1:**
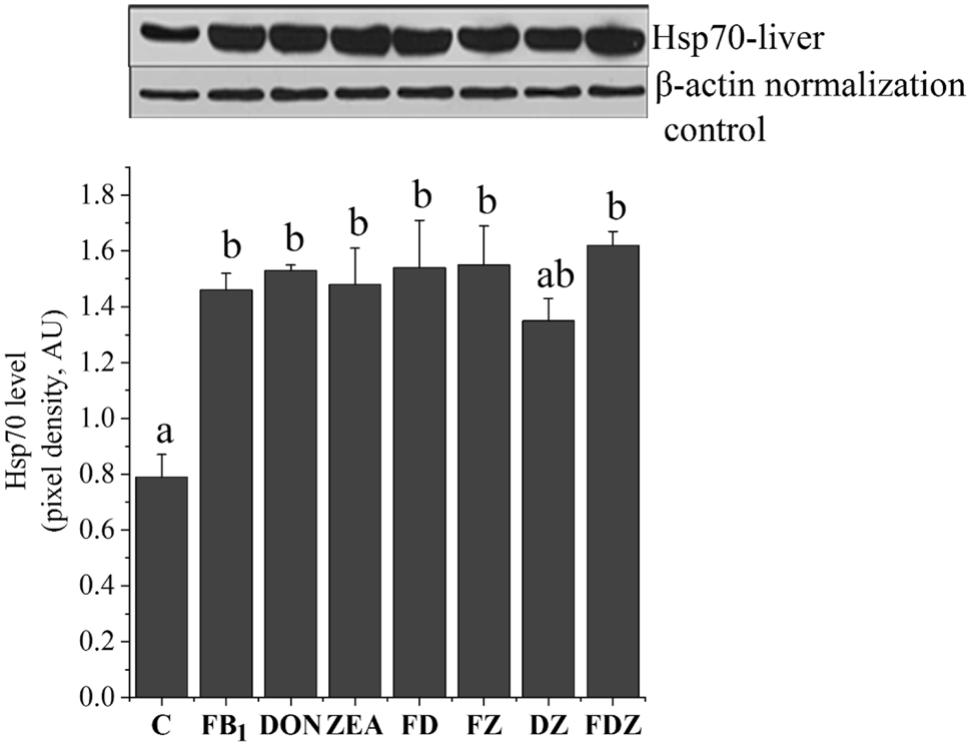
Hsp70 level in the liver tissue from different treatments, in which column and bars are representing the means and standard errors, respectively. The different letters (**a**, **b**, and **ab**) above the standard error bars indicate a significant difference at the *P* < 0.05 level. β-actin served as normalization control (42 kDa)

As a result of the toxin administration (Fig. 1), except in the DZ group, the Hsp70 level increased significantly (*P* < 0.05) in the liver, as compared with the control. Among the different toxin groups, the DON, FD, FZ and FDZ were significantly higher (*P* < 0.01) than the control.

When the mycotoxins’ interaction in liver was investigated, additive interactions detected in the binary doses, namely the FD and DZ group. The FDZ interaction revealed on the antagonistic outcome, in which the observed effect was less than the calculated expected value.

### Kidney Hsp70 levels

**Fig. 2:**
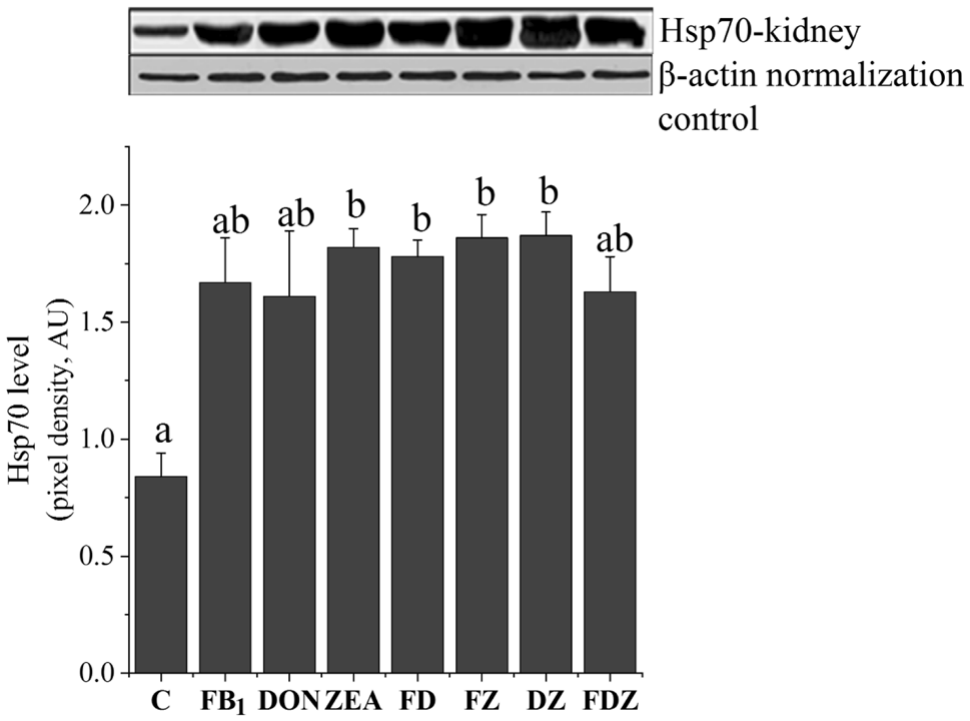
Hsp70 level in the kidney tissue from different treatments, in which column and bars are representing the means and standard errors, respectively. The different letters (**a**, **b**, and **ab**) above the standard error bars indicate a significant difference at the *P* < 0.05 level. β-actin served as normalization control (42 kDa)

We found several treatments to increase the Hsp70 level (*P* < 0.05), as compared with the control (Fig. 2). These treatments were individual (ZEN), binary (FD, FZ, and DZ) treatments, but not the ternary treatment (FDZ; not responsive).

If no significant difference between the individual and combined mycotoxin treatments was found in the renal tissue (see Fig. 2), the Bliss independence calculation was not carried out.

### Lung Hsp70 levels

**Fig. 3:**
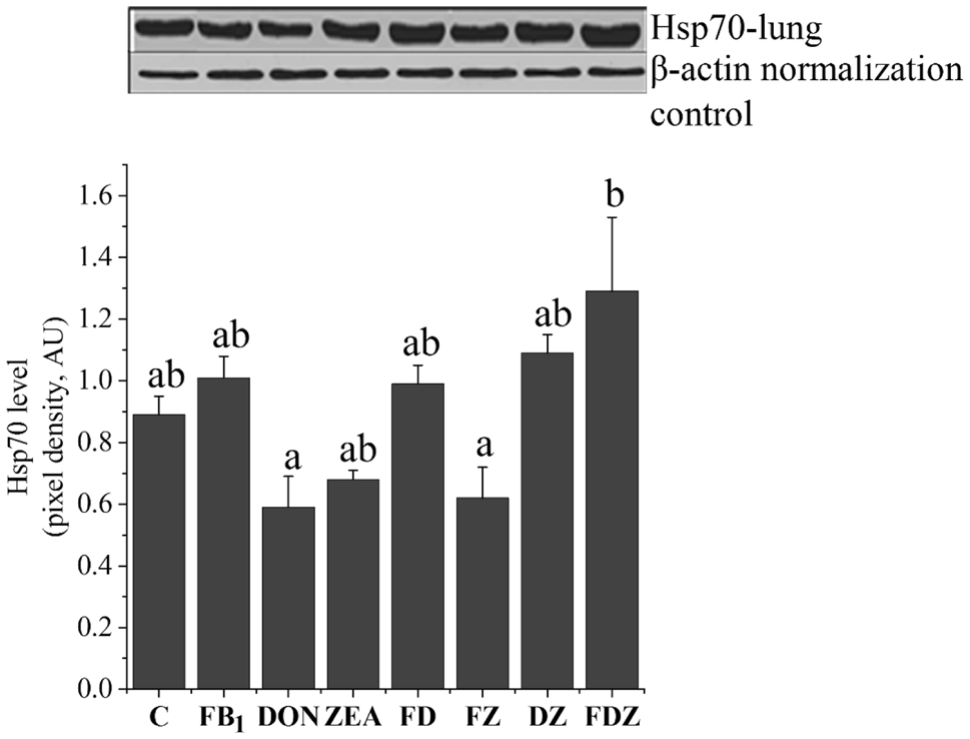
Hsp70 level in the lung tissue from different treatments, in which column and bars are representing the averages and standard errors, respectively. The different letters (**a**, **b**, and **ab**) above the standard error bars indicate a significant difference at the *P* < 0.05 level. β-actin served as normalization control (42 kDa)

When looking at the Hsp70 level in the lungs (Fig. 3), there was a significant increase (*P* < 0.05) in the FDZ animals, in comparison with individual DON and the binary DZ.

For the FDZ interaction assessment in the lung, the calculated values were lower than the observed values (antagonistic effect).

### Blood plasma clinical chemical parameters

The obtained results of plasma biochemical analysis are available in Table 1. The activity of ALT of rats exposed to the multitoxic treatment (FDZ) had significantly increased (*P* < 0.05), as compared with control. When compared with control, ZEN had decreased (*P* <  0.05) the total protein concentration. Other measured parameters were not responsive to any of the administered mycotoxins.

**Table 1:** Blood plasma clinical chemical parameters of the different groups, results represents mean ± **s**tandard deviation (M ± SD)

Parameter	Groups							
	C	FB1	DON	ZEN	FD	FZ	DZ	FDZ
	M ± SD	M ± SD	M ± SD	M ± SD	M ± SD	M ± SD	M ± SD	M ± SD
TP (g/L)	71.9 ± 0.50^b^	70.2 ± 0.27^ab^	68.2 ± 0.47^ab^	66.5 ± 0.84^a^	68.7 ± 0.26^ab^	68.2 ± 2.50^ab^	68.3 ± 0.10^ab^	69.9 ± 0.21^ab^
Albumin (g/L)	37.2 ± 0.76	36.3 ± 0.42	36.1 ± 0.31	35.4 ± 0.38	36.9 ± 0.59	36.1 ± 1.11	36.9 ± 0.52	36.6 ± 0.44
Urea (mmol/L)	10.5 ± 1.61	7.67 ± 0.27	9.87 ± 0.91	7.20 ± 0.42	7.33 ± 0.66	7.60 ± 0.65	8.33 ± 0.67	10.2 ± 1.98
AST (IU/L)	139 ± 1.86	106 ± 9.84	117 ± 5.90	106 ± 7.42	111 ± 3.84	122 ± 25.7	110 ± 5.36	132 ± 11.9
ALT (IU/L)	23.0 ± 4.72^a^	30.0 ± 1.53^ab^	26.3 ± 5.04^ab^	30.3 ± 0.88	30.0 ± 1.00^ab^	32.3 ± 4.48^ab^	31.0 ± 2.52^ab^	41.7 ± 4.06^b^

*TP* total protein, *AST* aspartate aminotransferase, *ALT* alanine aminotransferase

^a,b^different uppercase indices mean significant (*P* < 0.05) inter-group differences

In accordance with results obtained from Table 1, no interaction was detected between the individual and combined mycotoxin treatments.

### Plasma antioxidant parameters and lipid peroxidation

Oxidative stress–related results are shown out in Table 2. The plasma concentration of GSH significantly increased (*P* < 0.05) in the DON and ZEN groups, as compared with the control, FD, FZ and DZ. In comparison with the control, the GPx activity decreased significantly (*P* < 0.05) in the ZEN group, while no other treatment altered its level. We found the MDA concentration to increase (*P* < 0.05) in the DZ group, as compared with the FD group.

**Table 2:** Antioxidant and lipid peroxidation parameters in the blood plasma, results represent mean ± standard deviation (M ± SD)

Parameter	Groups							
	C	FB1	DON	ZEN	FD	FZ	DZ	FDZ
	M ± SD	M ± SD	M ± SD	M ± SD	M ± SD	M ± SD	M ± SD	M ± SD
GSH (μmol/g protein)	2.08 ± 0.16^a^	2.31 ± 0.06^ab^	2.27 ± 0.05^b^	2.57 ± 0.11^b^	1.93 ± 0.03^a^	2.06 ± 0.07^a^	2.05 ± 0.05^a^	2.27 ± 0.05^ab^
GPx (IU/g protein)	3.33 ± 0.09	3.06 ± 0.15	2.99 ± 0.09	2.65 ± 0.15	2.88 ± 0.15	2.93 ± 0.16	2.88 ± 0.08	2.83 ± 0.04
MDA (nmol/g protein)	12.4 ± 1.16^ab^	11.5 ± 0.65^ab^	12.1 ± 0.46^ab^	10.9 ± 0.18^ab^	9.44 ± 0.19^a^	11.0 ± 0.16^ab^	13.3 ± 1.58^b^	11.5 ± 0.16^ab^

*GSH* reduced glutathione, *GPx* glutathione peroxidase, *MDA* malondialdehyde

^a,b^different uppercase indices mean significant (*P* < 0.05) inter-group differences

With regard to the plasma GSH concentration, we found antagonistic interaction between mycotoxins for the binary (FD and FZ) and ternary (FDZ) mycotoxin doses. DZ yielded an antagonistic reaction in the plasma MDA concentration, whereas for FD, a synergistic relationship between toxins was found.

## Discussion

To the best of our knowledge, this is the first in vivo investigation on the individual and combined effects of *Fusarium* mycotoxins on the Hsp70 level. Mycotoxin effects vary between the different cells, depending on the toxin types and their plausible interactions. The role of oxidative damage in the toxicity is fundamentally important, as its origins and/or consequences determine the toxicity severity. The present study was part of a project which aims at the systematic assessment of the effects of the three mycotoxins on oxidative capacities in liver, kidney, and lung of the rat (Szabó et al. 2018).

### Liver Hsp70 levels

It is well recognised that numerous factors (i.e. hyperthermia, oxidative damage, physical injury or chemical stressors) could remarkably increase Hsp gene expressions (Fehrenbach and Northo 2001). The independent elevation in Hsp70 level from the redox system as induced by FB_1_ exposure has been reported in rat renal tissue (Kócsó et al. 2018, in vivo study) and in rat astrocyte line (Galvano et al. 2002), but not in rat liver. In our case, FB_1_ exposure increased the Hsp70 production in the rat liver. The possible scenario here is that the inhibition of ceramide synthesis by FB_1_ was the key factor behind the high Hsp70 expression in liver. According to Kondo et al. (2000), the intracellular ceramide generation was found to favour degradation of the Hsp70 mRNA.

A novelty of this study is the increase of Hsp70 with non-oxidative stress in the DON (a liver-damaging mycotoxin) treatment. DON has numerous modes of action rather than ROS generation to induce apoptosis or causing cellular stress, such as disruption of the cellular protein, DNA damage and/or different epigenetic mechanisms. We thus suggest that the damage of cellular proteins, a mode of action of DON elevated the Hsp70 expression. In BRL 3A rat liver cells, DON was more cytotoxic than other toxins and was associated with higher protein expression levels of p53 and Hsp70 (Sun et al. 2015). In the FDZ group, although the liver was not undergoing oxidative stress, hepatotoxicity was present. In a similar study design of ours by Szabó-Fodor et al. (2018), hepatotoxicity was proven (high lesion scores) after 5 days of DON and FDZ exposures (doses were 9, 16.5 and 12.75 µg of FB_1_, DON and ZEN/rat/day).

In our earlier published results (Szabó et al. 2018), high GSH levels and GPx activities were found in livers from ZEN-, FD- and FZ-treated groups. The potential increase in Hsp70 level for these groups might relate to the redox system activation. Hsp70 has many roles in maintaining cellular homeostasis, such as the protection against protein and lipids damage, increasing concentration of the intracellular calcium, sustaining ATP balance, and induction of antioxidant enzymes (Parsell and Lindquist 1994; Polla et al. 1996). ZEN is a highly oxidative mycotoxin, whereas Hsp70 expression is an early biomarker of ZEN toxicity. According to the in vivo study of Lee et al. (2013), ZEN was found to increase Hsp70 activity, GSH activity and MDA production in a dose-dependent manner in Chang liver cells. The authors also reported a decrease in Hsp70 production when the cytoprotective effect of N-acetylcysteine amide was applied. We proved no oxidative stress, suggesting Hsp70 expression is a complex mechanism controlled by numerous cellular events. A model proposed by Yamada and Nishimura (2008) includes that the inhibition of the Hsp90 ATPase stimulates the heat shock transcription factor that induces the expression of numerous HSP genes. ZEN has a similar radicicol structure and plays a role in the inhibition of Hsp90 ATPase activities (Torres Acosta et al. 2019), and consequently has the potential to alter the Hsp70 level.

### Kidney Hsp70 levels

The observed alterations in liver and kidney are closely reflecting the earlier possible stress conditions. In comparison with the liver, the kidney has shown slight differences in the Hsp70 expression (significant higher levels were in ZEN, FD, FZ and DZ groups). Up to date, the mechanism of the individual or combined mycotoxins in modifying the Hsp70 level is not fully understood. Some authors connect it to oxidative stress (El Golli-Bennour and Bacha 2011), while Hassen et al. (2007) proposed it is independent of cellular stress.

ZEN altered the Hsp70 level in the kidney, as well as in the liver. In monkey Vero kidney cell line exposed to citrinin, ZEN and T-2 toxins, the Hsp70 expression was dose-dependent, whereas the expression degree was decreased after vitamin E supplementation—a cytoprotective and antioxidant factor (El Golli et al. 2006; El Golli-Bennour et al. 2009). However, the cytoprotective effect of N-acetylcysteine amide could not improve the cell viability under sub-lethal ZEN dose (Lee et al. 2013). According to our earlier published results (Szabó et al. 2018), none of the Hsp70-altered groups has shown an alteration in the redox balance system, suggesting a non-oxidative molecular pathway.

The Hsp70 level did not show a deviation, although the MDA level was elevated in the kidney in the DON group (see Szabó et al. 2018). Despite DON is a non-pro-oxidant toxin, studies vary in conclusions. In vitro studies of Yang et al. (2014) and Zbyňovská et al. (2013) have documented the oxidative effect of DON on cells from human and swine, respectively. In contrast, a few studies showed that DON could not induce or had only negligible oxidative stress effects (El Golli-Bennour and Bacha 2011; Bensassi et al. 2009 and Korošec et al. 2008). The finding indicates that oxidative stress did not stimulate the Hsp70 production in the rat kidney, because it could be attributed to a delayed adaptation of the stress proteins and antioxidant defence system.

All binary treatments have increased Hsp70 level, but not the ternary treatment. The lack of increase in Hsp70-level of the FDZ group, unlike noted in the liver, may be attributed to the organ sensitivity to toxins and their interaction. Szabó-Fodor et al. (2018) have shown that intraperitoneal injection of FDZ to rats for 5 days could increase lesion scores in the hepatic tissue, but not in the renal tissue.

### Lung Hsp70 level

In the lungs, the only alteration (increase) found was in the FDZ group, as compared with the DON and FZ groups. When compared with results from liver and kidneys, the heat shock response to toxin combinations proved to be tissue and toxin specific. This refers to the fact that the generated stress proteins vary based on the species, cell/tissue type, stressor types, organ expression pattern and exposure time. At the moment, multitoxic interactions are not yet fully understood or well documented. The higher Hsp70 level in the lung of the FDZ group might result from toxicity and/or cellular injury. In the study of Szabó-Fodor et al. (2018), the FDZ exposure scored more lesions (pulmonary perivascular oedema). Once other treatments did not stimulate the Hsp70, their detailed discussion is void. Interestingly, a higher dose of FB_1_ (50 mg/kg diet) for 5 days was found to induce the lung Hsp70-production in rats (Kócsó et al. 2018), but not our lower dose.

### Plasma clinical chemical parameters

ALT is a liver-specific enzyme and its high activity in the blood plasma indicates the onset of hepatotoxicity in rats (Boyd 1983). Szabó-Fodor et al. (2018) have reported slight hepatotoxicity induced by FDZ exposure in rats by Szabó-Fodor et al. (2018). Authors have found histomorphological alterations in the liver and higher serum AST activity, although ALT activity was not responsive. AST is not a hepatotoxicity-specific biomarker (Tennant 1997), since its production takes place in the kidney, pancreas, skeletal muscle and erythrocytes. In our study, the AST was non-responsive in the FDZ group, unlike the ALT. This finding might result from the long exposure period (14 days), as compared with the 5 days treatment of Szabó-Fodor et al. (2018).

In this study, no nephrotoxicity was proven, or toxicity was negligible (could not compromise the albumin and urea concentrations). The decrease in total protein concentration caused by ZEN-exposure indicates the protein synthesis disruption in the liver. Other protein classes than albumin were probably the key players behind the total protein decrease. We speculate ZEN induced an immunosuppression effect that was reflected in the total protein concentration. In rats exposed to a 3 mg ZEN/kg diet, the immunotoxic effect of ZEN was recorded (Hueza et al. 2014). In the present study, no immunoglobulin test carried out, referring to the proof of this assumption is not possible in this study.

### Plasma antioxidant parameters and lipid peroxidation

GPx acts on the decomposition of H_2_O_2_ into H_2_O and O_2_ (Droge 2002). ZEN had modified the enzymatic antioxidant system in the blood plasma; decrease GPx. A similar ZEN-effect to ours on GPx level has been reported by Stadnik et al. (2009) and Zhou et al. (2015) in male Wistar rats and pregnant Sprague-Dawley rats, respectively. This decrease in GPx level indicates exhaustion of the enzymatic antioxidant capacity. The GSH is a co-substrate for GPx and increases to enforce GPx production (Trachootham et al. 2008). Individual toxins (DON and ZEN) have increased the extracellular GSH level, as compared with control. However, the increment in MDA level was not present in the two different treatments: DON and ZEN. The GSH and MDA findings, to an extent, refer indirectly to the activation of antioxidant defence mechanism and the efficiency of the redox system.

The DON and ZEN together increased MDA level, as compared to the FD (decreased). However, neither the GSH nor GPx was compromised, referring to less pronounced oxidative stress (Ali et al. 2019). The novelty of our study is that the individual and binary treatments could alter the cellular oxidative capacity, whereas the ternary *Fusarium* mycotoxins did not affect.

In accordance with Hsp70 results, oxidative damage was not always the key factors behind the Hsp70-level induction, suggestive of alternative molecular pathways. Therefore, further studies with larger population sizes are necessary to define these alternative molecular cascades in Hsp70-expression mediation, such as the pro-apoptotic factors besides the molecular chaperone studies.

Our study provided preliminary results on the interactive effects of *Fusarium*-toxins on Hsp70 production on rats. Mycotoxins could alter the Hsp70 production, whereas alteration varies according to the toxin types and their interactions. The induction in Hsp70 production by *Fusarium* mycotoxins was not always associated with oxidative stress, suggestive of alternative molecular events. The limitation of this study is the sample size, which may only reveal larger effects. Therefore, further studies are necessary to define those alternative molecular cascades in Hsp70-expression mediation, using a bigger population size.
